# Diversity patterns of free-living nematode assemblages in seagrass beds from the Cuban archipelago (Caribbean Sea)

**DOI:** 10.3897/BDJ.8.e58848

**Published:** 2020-12-23

**Authors:** Maickel Armenteros, Patricia Rodríguez-García, José Andrés Pérez-García, Adolfo Gracia

**Affiliations:** 1 Instituto de Ciencias del Mar y Limnología, Universidad Nacional Autónoma de México, México, Mexico Instituto de Ciencias del Mar y Limnología, Universidad Nacional Autónoma de México México Mexico; 2 Centro de Investigaciones Marinas, Universidad de La Habana, Habana, Cuba Centro de Investigaciones Marinas, Universidad de La Habana Habana Cuba

**Keywords:** richness, β-diversity, meiofauna, spatial patterns, distance decay of similarity, Caribbean Sea

## Abstract

Diversity patterns of free-living marine nematodes in tropical seagrass beds are understudied. Here, we describe the species richness and assemblage composition of nematodes in 13 seagrass sites covering the whole Cuban archipelago. Nematodes were collected from *Thalassia testudinum* seagrass beds and identified to species level. We provide a checklist of nematode species from seagrass beds. The species richness of nematode assemblages is high, with 215 species, 138 genus, 35 families, seven orders and two classes. That γ-diversity is higher than other studies and points to seagrass beds as diversity hotspots of free-living marine nematodes. Local species richness in seagrass bed sites is about 57 ± 17 species and broadly similar across the sites, despite the environmental heterogeneity. The geographical distance plays a weak, but significant, role on the decay of similarity likely affected by limited dispersal of nematodes. The pairwise similarity values, related to poor-coloniser nematodes, were twice more affected by the distance than those related to good-colonisers, possibly due to differential success of transport and settlement.

## Introduction

Nematodes constitute the fourth most diverse phylum of metazoans on Earth, after Arthropoda, Mollusca and Platyhelminthes (Zhang 2013). The number of accepted species of free-living marine nematodes is about 6219 species (Bezerra et al. 2020), but the percentage of known species is as low as 12% (Appeltans et al. 2012). This gap in knowledge is even larger in tropical regions since there are many more studies on nematode diversity in Europe and North America compared with Africa and Central America, despite tropical ecosystems harbouring some of the most diverse habitats on the Earth, such as corals reefs (Cote and Knowlton 2014) and seagrass beds (Duffy et al. 2014).

Seagrass beds, when compared with unvegetated adjacent habitats, harbour larger nematode species richness (Ruiz-Abierno and Armenteros 2017) and different species composition (Fonseca et al. 2011). However, few studies about nematode diversity in tropical/subtropical seagrass beds have been undertaken in comparison with intertidal and estuarine systems. In tropical seagrass beds, nematode assemblages have been studied by Hopper and Meyers (1967a), Hopper and Meyers (1967b), Ndaro and Olafsson (1999), Fisher (2003), Fisher and Sheaves (2003) and Liao et al. (2015). In temperate seagrass beds, other studies on nematode diversity have described relationships with food availability in *Posidonia* beds (Danovaro and Gambi 2002) and the assemblage response to a collapse of *Zostera* beds (Materatski et al. 2016, Materatski et al. 2018, Materatski et al. 2015, Branco et al. 2018). Seagrass beds provide an array of advantageous conditions to the meiofauna (and nematodes), such as physical protection against re-suspension, food availability derived from seagrass production and diverse microhabitats (Bell et al. 1984, Decho et al. 1985). This combination of sedimentary conditions/resources provides intermediate levels of resource availability and physical disturbance which, in turn, promotes the meiofaunal richness (Armenteros et al. 2019).

The difference patterns in species composition across samples, also termed as β-diversity, is a central theme in community ecology (Anderson et al. 2011). One pattern of β-diversity, suitable to explore in our study, is the distance decay of similarity (DDS, Nekola and White 1999). For meiofauna, patterns of β-diversity have been explained by a combination of niche assembly and dispersal processes (Giere 2009). Niche assembly processes have been the subject of many studies relating the nematode assemblage structure with the environment. Some of these studies explicitly addressed the niche breadth of nematodes (e.g. Wu et al. 2019) and other related assemblage structure with environmental factors looking for ecological drivers [see Heip et al. (1985) and Moens et al. (2014) for reviews]. Nematodes have the ability to effectively disperse at small scales (< 10 m) by both active swimming and passive transport (Ullberg and Ólafsson 2003, Thomas and Lana 2011). Dispersal of marine nematodes is substantial at scales of 10–100 km as indicated by genetic data (Derycke et al. 2013). A recent review (Ptatscheck and Traunspurger 2020) concluded that nematodes are effective colonisers due to the diversity of dispersal modes, continuous immigration and rapid reproduction.

In addition to taxonomic diversity, the functional diversity of assemblages can be addressed on the basis of biological traits. These traits refer to morphological, physiological or phenological features, measurable at the individual level (Violle et al. 2007). The most used biological traits of marine nematodes have been the trophic group and the coloniser/persister ability (Schratzberger et al. 2007, Armenteros et al. 2009, Alves et al. 2014). The trophic classification was proposed by Wieser (1953), based on the structure of stoma and, later, refined by Moens et al. (2004). The c-p classification was proposed as an arbitrary scale to measure the ability to colonise/persist of nematodes, based on food preference, gonad size, metabolic rate, mobility and occurrence of larva dauer (Bongers 1990, Bongers et al. 1991).

In this study, we contribute to the knowledge of the diversity of free-living marine nematodes in seagrass beds from the Cuban archipelago, one of the hotspots of diversity in the Caribbean Sea. Therefore, the aims of our research are:

(1) Quantify the species richness of free-living marine nematodes from seagrass beds at the scale of a single site (α-diversity) and of the whole archipelago (γ-diversity). We provide a checklist of species that accounts for the γ-diversity of Cuban seagrass beds; and

(2) Explore the β-diversity patterns and the effects of two potential environmental drivers: the geographical distance (c.f. distance decay of similarity) between the studied sites and the differential oceanic exposure of the sites (lagoon versus open shelf). In addition, the analysis of two biological traits of nematodes (trophic group and colonising capacity) adds another dimension to the analysis of β-diversity.

## Material and methods

### Study sites

**Figure 1.**
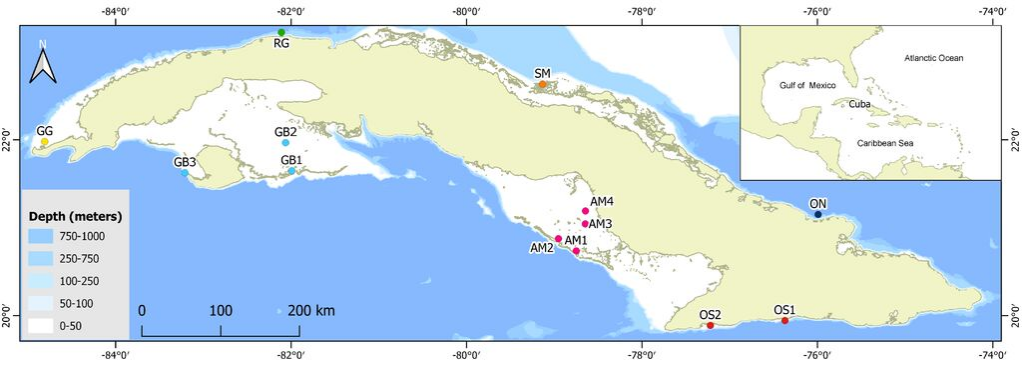
Map of the Cuban archipelago indicating the 13 sampled sites in seagrass beds. Dot colours indicate different areas in the Cuban shelf.

We sampled 13 sites located in extensive seagrass beds (at least 1 km^2^ of extension) in seven areas around the Cuban archipelago (Fig. 1 and Table 1). Six sites were exposed to the direct influence of oceanic waters (i.e. relatively close to the shelf border and no coral reef crest separating the bed from the shelf border): GG, RG, ON, OS1, OS2 and GB3. The other seven sites were separated from open waters by chains of cays and coral reef crests (i.e. sheltered): SM, AM1, AM2, AM3, AM4, GB1 and GB2. We ensured the accuracy of this classification on the basis of *in situ* observations of the sediment type (e.g. silty or sandy), bottom slope and biota that may indicate physical disturbance (e.g. gorgonians, sponges). The seagrass beds were composed mainly of turtle grass (*Thalassia testudinum*) in soft bottoms with depth range from 1 to 5 m.

### Sampling

We could not sample the whole extension of the archipelago in a single expedition; actually, sampling events were done within an interval of five years (see dates in Table 1) in seven expeditions with different goals. This is the reason for small variations in the field protocol, such as the number of collected cores or the use of preserving agent. Sediment cores (4–8 per site) of internal diameter 2.5 cm were collected by SCUBA divers in an area of ca. 36 m^2^ within each site and at a depth inside the sediment of 6 cm. The sediment was preserved on board in either 95% ethanol or 10% formalin.

Temperature, salinity and dissolved oxygen (DO) were measured *in situ* (but not at all sites) at approximately 10 cm from the bottom using an oceanographic Hydrolab multiprobe 4a instrument. Samples of the uppermost 3-cm layer of sediments were taken with a 250-ml propylene container for the measurement of grain size and total organic matter (TOM). Grain size was determined with the gravimetric method using a standard sieve column and an analytical balance (Loring and Rantala 1992) and was expressed as the percentages of mud (silt + clay, < 63 µm) and sand (> 63 µm). TOM was determined by the gravimetric method, measuring the loss of weight on ignition at 450°C for eight hours (Heiri et al. 2001).

The geographical distance between sites was calculated as the shortest distance across the sea to allow for potential dispersal by currents; i.e. no land barriers were crossed. Distances were calculated in the software OpenCPN 4.2.0 with nautical charts provided by GEOCUBA Nautical Cartography Agency.

### Processing of samples

In the laboratory, sediment was sieved with filtered water (32 µm) through a nested set of sieves of 45 and 500 µm of mesh size to separate the fractions of meiofauna and macrofauna, respectively. The material retained in the sieves was preserved in ethanol 70% (Evans and Paulay 2012) and both fractions were analysed to extract the nematodes. The sediment containing the organisms was mixed gently with a solution of sugar and water (density 1.15 g cm^-3^) following the procedure in Armenteros et al. (2008). This procedure allows the extraction of the organisms from the sediment by difference of density (i.e. sediment settles on the bottom and organisms float on the surface of the supernatant). The procedure was repeated three times, the supernatant being concentrated in a volume of ca. 25 ml and observed under a stereomicroscope Olympus SZX7 with magnification between 15 and 115x. All the nematodes in the samples were picked up with a sleeved needle ending in a hook and preserved; however, only a subset of nematodes were included in this study because other individuals were diverted for DNA analyses. We collected between 100 and 400 nematodes, the first to be observed in the counting chambers (i.e. no size or shape bias) and preserved them in 10% formalin. This splitting of the samples prevented a quantitative assessment of nematode abundance; so we necessarily focused on a nematode subset and not on the whole assemblage. Selected nematodes were mounted in permanent glass slides for microscopy, following the technique in Vincx (1996).

Nematodes were identified to the lowest possible taxonomic level, following the taxonomic literature, such as those by Platt and Warwick (1983), Platt and Warwick (1988), Warwick et al. (1998) and Schmidt-Rhaesa (2014). In many cases, the name of some species could not be assigned with reasonable accuracy because there were single individuals and/or they did not match with any described species within the genus (i.e. likely a new species); therefore, these species were referred as sp. in the text. If more than one morphospecies were recorded within a same genus, they were named with a number (i.e. sp. 1, sp. 2). For some complex genera with very small-sized nematodes and those, such as *Desmoscolex* and *Tricoma*, we could not discriminate beyond doubt between species and we preferred to group them as spp. (i.e. more than one species within the genus).

Nematodes were classified according to two different functional traits (feeding groups and coloniser/persister ability) (Suppl. material 1). The classification by Wieser (1953) of nematodes in four trophic categories, based on the morphology of the buccal cavity, was used: selective deposit feeders (1A), non-selective deposit feeders (1B), epigrowth feeders (2A) and predators/omnivores (2B). We also used the classification of nematodes, based on the coloniser/persister ability (Bongers 1990, Bongers et al. 1991) from c-p 1 (best colonisers) to c-p 5 (bad colonisers).

### Data analyses

Accumulation curves of richness versus individuals were built in the software EstimateS 9.0 (Colwell 2013). Richness was assessed using the observed number of species and the non-parametric Chao 2 estimator that accounts for the unseen taxa. The observed number of species and the rarefied richness for 100 specimens were calculated for each site. The associated 0.95 confidence intervals (CI) were calculated, based on 100 permutation in EstimateS 9.0. Overlapping of CIs was used as a relaxed indication of non-significant differences between sites. The association between richness and abundance across all the sites was calculated with the Spearman rank correlation coefficient (R_S_).

We used the complement of the Sorensen Similarity Index as a measure of β-diversity (i.e. 100 – Sorensen) between pairs of sites. Based on the triangular matrix of similarities, we did a numerical ordination of the sites by non-metric multidimensional scaling (NMDS) to visualise potential patterns in β-diversity. We used the Sorensen Index, which relies on presence/absence data, because only a subset of nematodes in the samples were analysed. Statistical significance between the oceanographic regimes (two states: exposed versus sheltered) was undertaken using an Analysis of Similarity (ANOSIM) in the software PRIMER 6.1.15 (Clarke and Gorley 2006).

An analysis of linear regression was carried out to test the dependence of the Sorensen pairwise similarity with the geographical distance. Here, we used the Sorensen similarity measure (instead of dissimilarity) because previous studies have tested the distance decay model using similarity measures. A Mantel-type test of significance, using a non-parametric approach, was made using the routine RELATE in the software PRIMER 6.1.15 to test if both triangular matrices were significantly related.

## Data resources

The data underpinning the analysis reported in this paper are deposited at GBIF, the Global Biodiversity Information Facility, http://ipt.pensoft.net/resource.do?r=xxxxxx

## Results

### Abiotic factors

Salinity and dissolved oxygen in the water column had a narrow range (35–37 PSU and 6.1–8.6 mg l^-1^, respectively), typical of marine and well-oxygenated shallow waters (Table 1). The fraction of fine sediment (grain size < 63 µm) in sediment had broad differences between exposed (mean: 6.5%) and sheltered sites (mean: 55%). The content of organic matter was also broadly different with exposed sites having poorer content of carbon in sediments (mean: 6%) compared to sheltered sites (mean: 17%) (Table 1).

### Species richness

**Figure 2.**
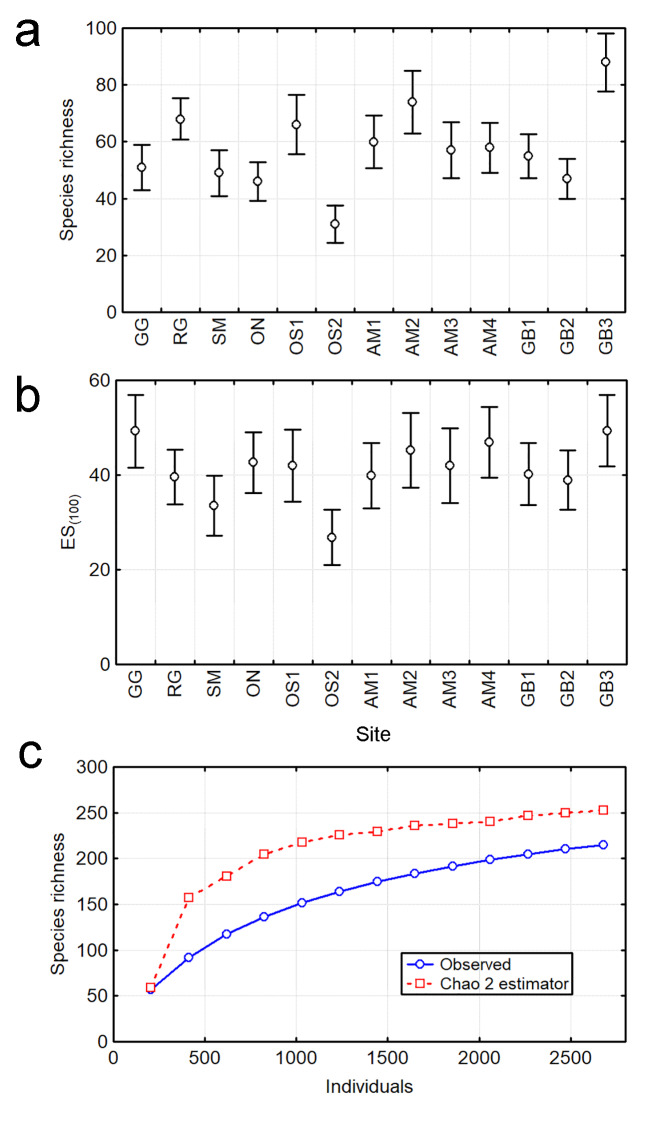
Species richness of free-living marine nematode assemblages in seagrass beds. (a) Observed species richness per site (α-diversity) with 0.95 confidence intervals. (b) Expected number of species (ES) rarefied to a sample of 100 nematodes per site. (c) Accumulation curves of observed species richness and the non-parametric Chao 2 estimator with all sites combined (γ-diversity).

We identified 2678 nematodes belonging to 215 species, 138 genera, 34 families, seven orders and two classes. The observed species richness at local scale (α-diversity) had a median (± interquartile range, n = 13) of 57 ± 17 species (range: 31–88 species). Species richness did not show significant differences between most of the sites as indicated by the broad overlapping of the confidence intervals; but GB3 and OS2 had the lowest and highest values of α-diversity, respectively (Fig. 2a). Since species richness was strongly influenced by the number of identified specimens (Spearman correlation, R_S_ = 0.82, p < 0.001, n = 13), we calculated the expected number of species (ES) for a sample size of 100 specimens, using rarefaction. ES_(100)_ was not correlated with the abundance (R_S_ = 0.05, p = 0.87, n = 13). The median ES_(100)_ was 42 ± 6 species (range: 27–49 species). The broad overlapping of the 0.95 confidence intervals for ES_(100)_ suggests no significant differences in the species richness between sites; albeit the significant differences remained between GB3 and OS2 (Fig. 2b).

The species richness at regional scale (γ-diversity) of free-living marine nematodes in seagrass beds was estimated from the combination of the 13 studied sites. The curve of accumulation of observed species richness did not approach to an asymptote (Fig. 2c) and the total richness (± SD) was 215 ± 6 species (0.95 CI: 204–226 species). However, the curve of Chao 2 non-parametric estimator approached to an asymptote and the estimated species richness was 253 ± 13 species (0.95 CI: 235–287 species). The checklist of species contributing to the regional inventory and their occurrence across the 13 sites is given as supplementary material (Suppl. material 2).

The more abundant trophic groups (median ± interquartile range) were predator/omnivores (2B, 35% ± 15%) and epigrowth feeders (2A, 34% ± 7%). The third most abundant trophic group was non-selective deposit feeders (1B, 19% ± 11%) and the less abundant was selective deposit feeders (1A, 14% ± 5%). According to the coloniser/persister scale, the nematodes with intermediate colonising ability were the most abundant (c-p 3, 47% ± 13%), followed by nematodes with high colonising ability (c-p 2, 40% ± 13%). Nematodes with low colonising abilities had lower abundance (c-p 4, 12% ± 4%) and nematodes, with the lowest colonising ability, were the least abundant (c-p 5, 0.5% ± 0.9%).

### Assemblage composition and β-diversity

The dominance was moderate with only 23 species (11% of total richness) accounting for 51% of the total accumulated abundance. The most abundant species were *Paradesmodora immersa* Wieser, 1954 (4%); *Desmodora pontica* Filipjev, 1922 (4%); *Viscosia abyssorum* (Allgén, 1933) (3%); *Dorylaimopsis punctata* Ditlevsen, 1918 (3%); *Daptonema* sp. (3%); *Marylynnia* sp. (3%), *Euchromadora vulgaris* Bastian, 1865 (3%); *Halichoanolaimus chordiurus* Gerlach, 1955 (3%); and *Zalonema ditlevseni* (Micoletzky, 1922) (3%). These species belonged to five orders and seven families widely distributed in marine habitats, namely: Chromadoridae, Comesomatidae, Cyatholaimidae, Desmodoridae, Oncholaimidae, Selachinematidae and Xyalidae.

**Figure 3.**
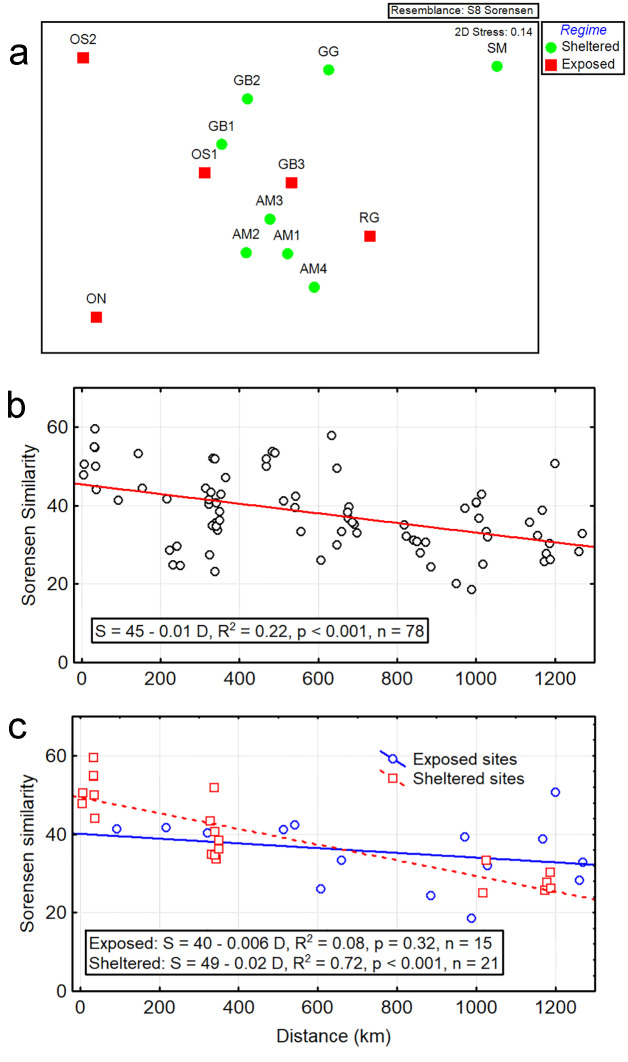
Species composition of free-living marine nematode assemblages in seagrass beds. (a) Ordination of 13 sites in seagrass beds of the Cuban archipelago, based on presence/absence of free-living nematode species. (b) Relationships between pairwise Sorensen similarity and geographical distance. (c) Relationships between Sorensen similarity and geographical distance with sites separated by the oceanographic regime.

The values of pairwise dissimilarity (β-diversity) between sites ranged from 40 to 67% with an average of 55%. The ordination of the sites, based on the presence/absence of species, did not indicate any clustering of sites, based on the geographical area or oceanographic regime (i.e. exposed vs. sheltered) (Fig. 3a). The multivariate assemblage structure between regime conditions was not significantly different (ANOSIM, R = 0.08, p = 0.25, 999 permutations).

Geographical distance may be a driver of β-diversity, namely, the model distance decay of similarity (DDS). To explore the adjustment of DDS model of our data, we computed 78 shortest geographical distances amongst the 13 sites. The geographical distance amongst sites had a median of 599 km (range: 5–1269 km). The Sorensen pairwise similarity between sites was significantly, but weakly, related with the geographical distance (linear regression, slope = -0.01 ± 0.003, p < 0.001, R^2^ = 0.22, n = 78) (Fig. 3b). A non-parametric approach using a Mantel Test indicated that both triangular matrices (Sorensen similarity and geographical distance) were significantly associated (RELATE, Rho = 0.46, p = 0.004, 999 permutations).

We also explored the relationships between Sorensen similarity with geographical distance independently for exposed and sheltered sites. Exposed sites lacked a DDS as indicated by the non-significant relationship between Sorensen similarity and geographical distance. However, sheltered sites had a DDS relationship with a significant relationship between similarity and distance (Fig. 3c and Table 2).

**Figure 4.**
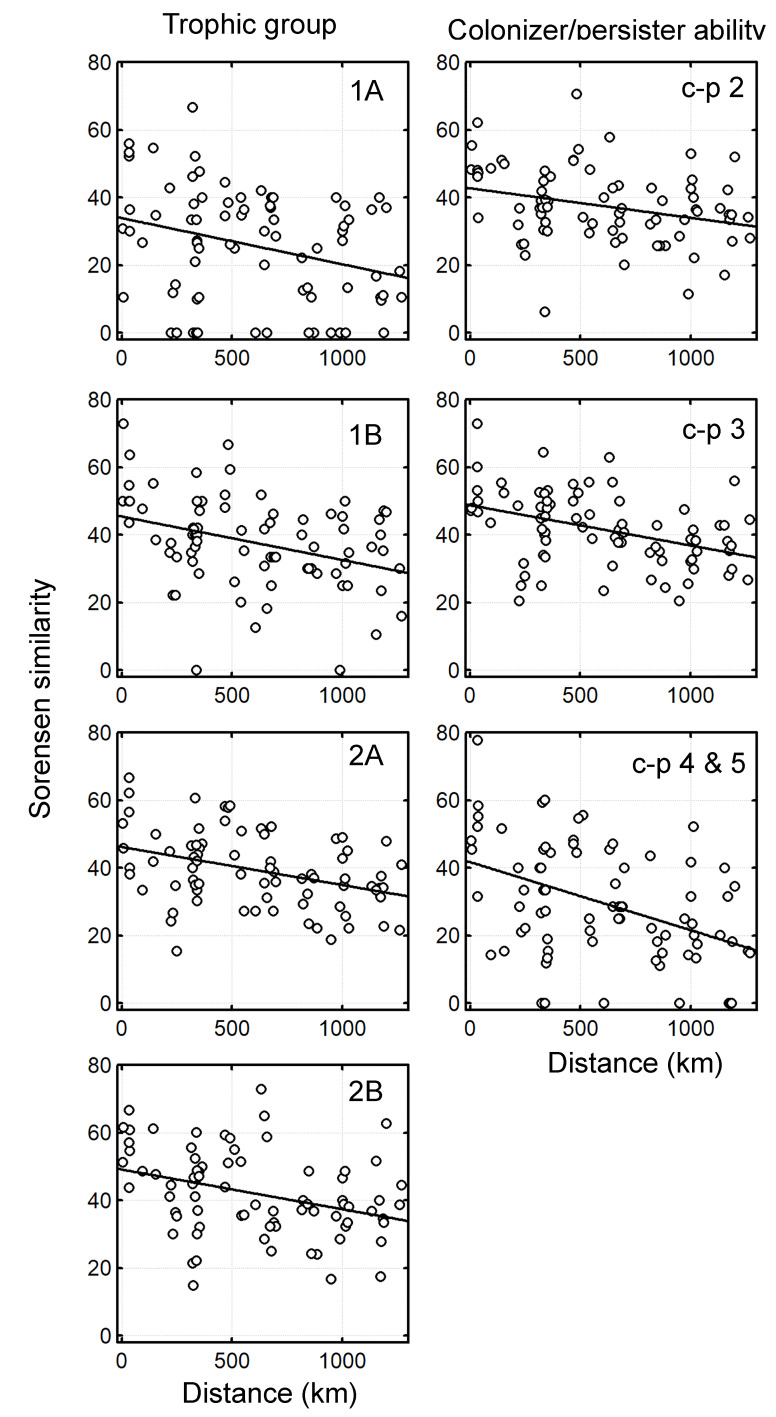
The distance decay of similarity for pairwise similarity values (Sorensen Index) of free-living marine nematode assemblages and shortest geographical distance between sites. Sorensen similarities are based on matrices of functional traits. Left column: Trophic groups (selective deposit feeders = 1A, non-selective deposit feeders = 1B, epigrowth feeders = 2A and predator/omnivores = 2B). Right column: Coloniser/persister scale (c-p 2 good coloniser/poor persister to c-p 5 bad coloniser/good persister).

The DDS occurred for the four tested trophic groups: deposit feeders (1A and 1B), epigrowth feeders (2A) and predator/omnivore (2B) (Fig. 4, left column and Table 2). The slope of the relationship similarity-distance for these groups was statistically significant and similar in magnitude with a decay of ca. 1% of similarity for each 100 km. The DDS was also significant for all the categories of nematodes classified after the coloniser/persister scale (Fig. 4, right column and Table 2). However, the slope of poor colonisers (i.e. c-p 4 & 5) was two times greater (ca. 2% change of similarity each 100 km) than good colonisers (ca. 1% change each 100 km).

## Discussion

### Species richness

The γ-diversity of nematode assemblages was high as expected in seagrass beds. We have reported higher species richness than other studies in tropical seagrass beds [e.g. 100 species in Hopper and Meyers (1967b) from four sites, 152 species in Fisher and Sheaves (2003) from four sites]. Most of the studies of nematode diversity in seagrass beds have used genus as the lowest taxonomic level. The diversity at genus level reported in our study (138 genera) is higher than other reports in temperate seagrass beds [e.g. 88 genera in Danovaro and Gambi (2002) within a single *Posidonia* bed, 58 genera in Materatski et al. (2015) from two sites]; and also higher than other estimates in tropical seagrass beds [e.g. 100 genera in Ndaro and Olafsson (1999)from three sites, 63 genera in Liao et al. (2015) within a single *Thalassia* bed]. The most plausible explanation is the large spatial coverage of our sampling scheme that included 13 sites in seagrass beds from all the sub-regions in the Cuban archipelago (i.e. separated in median 599 km). Another factor that could promote the high species richness is the variety of oceanographic regimes in the sampling sites, ranging from widely exposed (OS1 and OS2) to well-sheltered (AM1 and AM2). The non-asymptotic shape of the curves of accumulated number of species suggests that there is still undiscovered diversity. The non-parametric Chao 2 estimator indicates an as yet unseen diversity as high as 253 species that can be interpreted as the lower bound of diversity (Gotelli and Colwell 2011) in the studied seagrass beds.

The significant differences in α-diversity between sites could be explained by differences in the number of identified specimens and/or environmental conditions at local scale. However, when we standardised to an equal level of abundance, the species richness was the same across most of the sites. It suggests that the seagrass beds around the Cuban archipelago harbour a rather similar species richness, despite the environmental heterogeneity. This is consistent with the lack of clustering of the samples, based on assemblage species composition, by geographical area or exposure to oceanic influence.

The recorded broad phylogenetic scope of the nine dominant nematode species in our study (belonging to five orders and seven families), high species richness and even occurrence of all trophic groups, reflect the combination of: (i) heterogeneous composition of food sources (Danovaro and Gambi 2002), (ii) the provision of sheltered environment by the seagrass blades (Leduc and Probert 2011) and (iii) the diversity of microhabitats in seagrass beds (Hall and Bell 1993). This combination of enhancing factors supports the view that seagrass beds are diversity hotspots for free-living marine nematodes with higher richness, compared to other habitats (Armenteros et al. 2019).

### β-diversity

The geographical distance influenced the pairwise similarity in the studied assemblages, albeit with weak effect. The DDS can be caused by either a decrease in environmental similarity with distance or by limits to dispersal and niche breadth differences amongst taxa (Nekola and White 1999). The separation between seagrass beds in our study (median 599 km) likely constitutes limits to dispersal. Distances in the order of more than 100 km constitute limits to the dispersal of marine nematodes as evidenced from genetic (Derycke et al. 2013) and ecological data (Pérez-García et al. 2019). However, aquatic nematodes may disperse to long distances by the combination of diverse modes of dispersal (e.g. passive dispersal by currents, zoochory) with rapid reproduction (Ptatscheck and Traunspurger 2020). The differences in the DDS pattern between exposed and sheltered sites suggest that dispersal by hydrodynamics plays a significant role. Stronger hydrodynamics in exposed sites putatively resulted in a weaker DDS when compared with sheltered sites where weaker hydrodynamics may pose limits to the passive dispersal. However, alternative explanations, such as the difference in the pool of organic carbon in sediments (lower in exposed sites) and/or grain size (higher in exposed sites), cannot be ruled out and deserve further study using abundance data.

The DDS patterns occurred in similar rates for the four trophic groups (i.e. diminution of similarity with the increase in geographical distance). According to Danovaro and Gambi (2002), the nematode trophic group composition in seagrass beds was mainly determined by the quality and quantity of available food in sediments. In our study, it is unclear if DDS patterns, based on trophic groups, were affected by the distance itself or was mirroring the decay of similarity of species composition. Two further issues, related to the use of trophic group classification, could affect our results. First, Wieser’s classification does not characterise effectively the high feeding selectivity and flexibility of nematodes (Moens et al. 2014). Second, swimming behaviour of the species, even within the same Wieser’s feeding group, affects the susceptibility to passive re-suspension as indicated by experimental evidence (Thomas and Lana 2011).

The differential colonising ability of nematodes significantly affected the distance decay of similarity. The similarity pattern of poor coloniser nematodes (c-p 4 and 5) was twice as much affected by the geographical distance than the good colonisers (c-p 2 and 3). This suggests that the organismal features used for c-p scale, namely, generation time, reproduction rate and body size (Bongers 1990, Bongers et al. 1991) may affect the distance decay patterns of nematodes. Likely, large body-sized nematodes with low generation times, few offspring and low metabolic rate (e.g. *Cylicolaimus magnus*, *Desmoscolex* spp., *Enoplus* sp., *Leptosomatum* sp. and *Polygastrophora maior*) were less effectively dispersed and/or settled with lower success to adjacent sites. On the other hand, good colonisers with small body size, producing many offspring and with higher metabolic rate, show a weaker distance decay likely due to more efficient dispersal by currents and/or successful settlement. Thus, according to Poulin (2003): “distance matters, but it is not always the key determinant of similarity”

### Concluding remarks

The regional species richness of free living nematode assemblages accounted for 215 species and 34 families. This γ-diversity was higher than other estimates from tropical and temperate regions and points to seagrass beds as diversity hotspots of free-living marine nematodes. Local species richness in seagrass sites was about 57 ± 17 species. The geographical distance played a weak, but significant, role in the decay of similarity and was likely affected by the limited dispersal of nematodes. Pairwise similarity of bad-coloniser nematodes was twice as much affected by distance than good-colonisers possibly due to differential success of transport and settlement.

## Supplementary Material

D75E46D0-99DC-552C-A682-39110ED12666 10.3897/BDJ.8.e58848.suppl1 Supplementary material 1 Classification of nematode species into c-p scale and trophic groups Data type Morphological and functional File: oo_481539.xlsx https://binary.pensoft.net/file/481539 Armenteros M, Rodríguez-García P, Pérez-García JA, Gracia A

3DF7FAA7-47FA-5378-BCDE-E896C06C5F3D 10.3897/BDJ.8.e58848.suppl2 Supplementary material 2 Counts of species of marine nematodes in 13 sites of seagrass beds from Cuban archipelago Data type Occurrence File: oo_455313.docx https://binary.pensoft.net/file/455313 Armenteros M, Rodríguez-García P, Pérez-García JA, Gracia A

## Figures and Tables

**Table 1.** Location and characteristics of the 13 sampling sites in seagrass beds from Cuban archipelago. DO = dissolved oxygen, TOM = total organic matter. Hyphens indicate no measures.

Site	Geographical area	Sampling date	Latitude (N) / Longitude (W)	Depth (m)	Salinity (PSU)	DO (mg l^-1^)	TOM (%)	Silt + clay (%)	Oceanic regime influence
GG	Gulf of Guanahacabibes	June 2014	22°00.582N, 84°48.783W	5	35	7.9	5	6	exposed
RG	Rincón de Guanabo	July 2015	23°10.621N, 82°05.951W	2	-	-	-	-	exposed
SM	Cayo Santa María	December 2018	22°39.358N, 79°04.224W	1	-	-	-	-	sheltered
ON	Oriente Norte	November 2014	21°12.329N, 76°14.201W	1	-	-	-	-	exposed
OS1	Oriente Sur	November 2014	19°57.909N, 76°19.478W	1	-	-	-	-	exposed
OS2	Oriente Sur	November 2014	19°54.573N, 77°12.045W	1	-	-	-	-	exposed
AM1	Gulf of Ana María	October 2013	20°48.861N, 78°52.983W	3	37	8.6	19	32	sheltered
AM2	Gulf of Ana María	October 2013	20°50.675N, 78°54.755W	3	36	7.8	20	87	sheltered
AM3	Gulf of Ana María	October 2013	21°05.299N, 78°43.592W	2	37	6.5	24	61	sheltered
AM4	Gulf of Ana María	October 2013	21°06.349N, 78°43.211W	2	37	6.6	20	47	sheltered
GB1	Gulf of Batabanó	May 2015	21°38.036N, 81°56.346W	1	37	6.1	9	55	sheltered
GB2	Gulf of Batabanó	May 2015	21°55.565N, 81°58.354W	5	36	8.2	10	46	sheltered
GB3	Gulf of Batabanó	February 2013	21°37.003N, 83°11.593W	2	35	7.0	7	7	exposed

**Table 2.** Testing the distance decay of similarity after oceanographic regime of the sites; and after the functional traits of free-living marine nematode assemblages. Parameters of the linear regression between the Sorensen Similarity Index and the geographical distance are given. Trophic groups are based on the structure of the buccal cavity after Weiser (1953) and coloniser/persister are based on a scale from good (c-p = 2) to poor (c-p = 5) coloniser. Scales 4 and 5 were summed. Asterisk (and bold) indicates that the slope is significantly different of zero.

Attribute/functional trait	Category/scale	R^2^	Slope	n
Oceanographic regime	Exposed	0.08	-0.006	15
	**Sheltered**	**0.72**	-0.02*	**21**
Trophic group	**Selective deposit feeders (1A)**	**0.09**	-0.01*	**78**
	**Non-selective deposit feeder (1B)**	**0.12**	-0.01*	**78**
	**Epigrowth feeder (2A)**	**0.14**	-0.01*	**78**
	**Predator/omnivore (2B)**	**0.12**	-0.01*	**78**
Coloniser/persister	**2**	**0.08**	-0.01*	**78**
	**3**	**0.17**	-0.01*	**78**
	**4+5**	**0.19**	-0.02*	**78**

## References

[B6406739] Alves A. S., Veríssimo H., Costa M. J., Marques J. C. (2014). Taxonomic resolution and Biological Traits Analysis (BTA) approaches in estuarine free-living nematodes. Estuarine, Coastal and Shelf Science.

[B6120549] Anderson M. J., Crist T. O., Chase J. M., Vellend M., Inouye B. D., Freestone A. L., Sanders N. J., Cornell H. V., Comita L. S., Davies K. F., Harrison S. P., Kraft N. J.B., Stegen J. C., Swenson N. G. (2011). Navigating the multiple meanings of diversity: a roadmap for the practicing ecologist. Ecology Letter.

[B6120569] Appeltans W., Ahyong S. T., Anderson G., Angel M. V., Artois T., Bailly N., Bamber R., Barber A., Bartsch I., Berta A. (2012). The magnitude of global marine species diversity. Current Biology.

[B6120594] Armenteros M., Pérez-García J. A., Pérez-Angulo A., Williams J. P. (2008). Efficiency of extraction of meiofauna from sandy and muddy marine sediments. Revista de Investigaciones Marinas.

[B6406727] Armenteros M., Ruiz-Abierno A., Fernández-Garcés R., Pérez-García J. A., Díaz-Asencio L., Vincx M., Decraemer W. (2009). Biodiversity patterns of free-living marine nematodes in a tropical bay: Cienfuegos, Caribbean Sea. Estuarine, Coastal and Shelf Science.

[B6120585] Armenteros M., Pérez-García J. A., Marzo-Pérez D., Rodríguez-García P. (2019). The influential role of the habitat on the diversity patterns of free-living aquatic nematode assemblages in the Cuban archipelago. Diversity.

[B6120612] Bell S. S., Walters K., Kern J. C. (1984). Meiofauna from seagrass habitats: A review and prospectus for future research. Estuaries.

[B6121085] Bezerra T. N., Decraemer W., Eisendle-Flöckner U., Hodda M., Holovachov O., Leduc D., Miljutin D., Mokievsky V., Peña Santiago R., Sharma J., Smol N., Tchesunov A., Venekey V., Zhao Z., Vanreusel A. Nemys: World Database of Nematodes. http://www.marinespecies.org.

[B6120621] Bongers T. (1990). The maturity index: an ecological measure of environmental disturbance based on nematode species composition. Oecologia.

[B6120630] Bongers T., Alkemade R., Yeates G. W. (1991). Interpretation of disturbance-induced maturity decrease in marine nematode assemblages by means of the Maturity Index. Marine Ecology Progress Series.

[B6120641] Branco J., Pedro S., Alves A. S., Ribeiro C., Materatski P., Pires R., Cacador I., Ado H. (2018). Natural recovery of Zostera noltii seagrass beds and benthic nematode assemblage responses to physical disturbance caused by traditional harvesting activities. Journal of Experimental Marine Biology and Ecology.

[B6120656] Clarke K. R., Gorley R. N. (2006). Primer v6: User manual/tutorial.

[B6120676] Colwell R. K. (2013). EstimateS: Statistical Estimation of Species Richness and Shared Species from Samples. http://purl.oclc.org/estimates.

[B6406670] Cote I. M., Knowlton N., Bertness M. D., Bruno J. F., Silliman B. R., Stachowicz J. J. (2014). Coral reef ecosystems. A decade of discoveries. Marine Community Ecology and Conservation.

[B6120686] Danovaro R., Gambi C. (2002). Biodiversity and trophic structure of nematode assemblages in seagrass systems: evidence for a coupling with changes in food availability. Marine Biology.

[B6120695] Decho A. W., Hummon W. D., Fleeger J. W. (1985). Meiofauna-sediment interactions around subtropical seagrass sediments using factor analysis. Journal of Marine Research.

[B6120705] Derycke S., Backeljau T., Moens T. (2013). Dispersal and gene flow in free-living marine nematodes. Frontiers in Zoology.

[B6406657] Duffy J. E., Hughes A. R., Moksnes P., Bertness M. D., Bruno J. F., Silliman B. R., Stachowicz J. J. (2014). Ecology of seagrass communities. Marine Community Ecology and Conservation.

[B6408090] Evans N., Paulay G., Kress W. J., Erickson D. L. (2012). Barcoding methods for invertebrates. DNA Barcodes: Methods and Protocols.

[B6120716] Fisher R. (2003). Spatial and temporal variations in nematode assemblages in tropical seagrass sediments. Hydrobiologia.

[B6120726] Fisher R., Sheaves M. J. (2003). Community structure and spatial variability of marine nematodes in tropical Australian pioneer seagrass meadows. Hydrobiologia.

[B6120736] Fonseca G., Hutchings P., Gallucci F. (2011). Meiobenthic communities of seagrass beds (Zostera capricorni) and unvegetated sediments along the coast of New South Wales, Australia. Estuarine, Coastal and Shelf Science.

[B6120745] Giere O. (2009). Meiobenthology. The microscopic motile fauna of aquatic sediments.

[B6120753] Gotelli N. J., Colwell R. K., Magurran A. E., McGill B. J. (2011). Estimating species richness. Biological Diversity. Frontiers in Measurement and Assessment.

[B6120767] Hall M. O., Bell S. S. (1993). Meiofauna on the seagrass Thalassia
testudinum: population characteristics of harpacticoids copepods and associations with algal epiphytes. Marine Biology.

[B6120777] Heip C., Vincx M., Vranken G. (1985). The ecology of marine nematodes. Oceanography and Marine Biology. An Annual Review.

[B6120788] Heiri O., Lotter A. F., Lemcke G. (2001). Loss on ignition as a method for estimating organic and carbonate content in sediments: Reproducibility and comparability of results. Journal of Paleolimnology.

[B6120798] Hopper B. E., Meyers S. P. (1967). Foliicolous marine nematodes on turtle grass, Thalassia
testudinum Knig, in Biscayne Bay, Florida. Bulletin of Marine Science.

[B6120808] Hopper B. E., Meyers S. P. (1967). Populations studies on benthic nematodes within a subtropical seagrass community. Marine Biology.

[B6120818] Leduc D., Probert P. K. (2011). Small-scale effect of intertidal seagrass (Zostera muelleri) on meiofaunal abundance, biomass, and nematode community structure. Journal of the Marine Biological Association of the United Kingdom.

[B6120828] Liao J. -X., Yeh H. -M., Mok H. -K. (2015). Meiofaunal communities in a tropical seagrass bed and adjacent unvegetated sediments with note on sufficient sample size for determining local diversity indices. Zoological Studies.

[B6120837] Loring D. H., Rantala R. T.T. (1992). Manual for the geochemical analyses of marine sediments and suspended particulate matter. Earth-Sciences Reviews.

[B6120865] Materatski P., Vafeiadou A. -M., Ribeiro R., Moens T., Adao H. (2015). A comparative analysis of benthic nematode assemblages from Zostera noltii beds before and after a major vegetation collapse. Estuarine, Coastal and Shelf Science.

[B6120856] Materatski P., Vafeiadou A. -M., Moens T., Adao H. (2016). Structural and functional composition of benthic nematode assemblages during a natural recovery process of Zostera noltii seagrass beds. Estuaries and Coasts.

[B6120846] Materatski P., Ribeiro R., MoreiraSantos M., Sousa J. P., Ado H. (2018). Nematode biomass and morphometric attributes as descriptors during a major Zostera noltii collapse. Marine Biology.

[B6120900] Moens T., Yeates G. W., De Ley P. (2004). Use of carbon and energy sources by nematodes. Nematology Monographs and Perspectives.

[B6120875] Moens T., Braeckman U., Derycke S., Fonseca G., Gallucci F., Gingold R., Guilini K., Ingels J., Leduc D., Vanaverbeke J., Van Colen C., Vanreusel A., Vincx M., Schmidt-Rhaesa A. (2014). Ecology of free-living marine nematodes. Nematoda.

[B6120909] Ndaro S. G.M., Olafsson E. (1999). Soft-bottom fauna with emphasis on nematode assemblage structure in a tropical intertidal lagoon in Zanzibar, eastern Africa: I. Spatial variability. Hydrobiologia.

[B6120923] Nekola J. C., White P. S. (1999). The distance decay of similarity in biogeography and ecology. Journal of Biogeography.

[B6120932] Pérez-García J. A., Marzo-Pérez D., Armenteros M. (2019). Spatial scale influences diversity patterns of free-living nematode assemblages in coral degradation zones from the Caribbean Sea. Marine Biodiversity.

[B6120941] Platt H. M., Warwick R. M. (1983). Free-living marine nematodes. Part I. British Enoplids.

[B6120949] Platt H. M., Warwick R. M. (1988). Free-living marine nematodes. Part II. British Chromadorids.

[B6120959] Poulin R. (2003). The decay of similarity with geographical distance in parasite communities of vertebrate hosts. Journal of Biogeography.

[B6120968] Ptatscheck C., Traunspurger W. (2020). The ability to get everywhere: dispersal modes of free-living, aquatic nematodes. Hydrobiologia.

[B6120977] Ruiz-Abierno A., Armenteros M. (2017). Coral reef habitats strongly influence the diversity of macro and meiobenthos in the Caribbean. Marine Biodiversity.

[B6120986] Schmidt-Rhaesa A. (2014). Nematoda.

[B6406718] Schratzberger M., Warr K., Rogers S. I. (2007). Functional diversity of nematode communities in the southwestern North Sea. Marine Environmental Research.

[B6120994] Thomas M. C., Lana P. C. (2011). A new look into the small-scale dispersal of free-living marine nematodes. Zoologia.

[B6121003] Ullberg J., Ólafsson E. (2003). Free-living marine nematodes actively choose habitat when descending from the water column. Marine Ecology Progress Series.

[B6121012] Vincx M., Hall G. S. (1996). Meiofauna in marine and freshwater sediments. Methods for the examination of organismal diversity in soils and sediments.

[B6406688] Violle Cyrille, Navas Marie-Laure, Vile Denis, Kazakou Elena, Fortunel Claire, Hummel Irène, Garnier Eric (2007). Let the concept of trait be functional!. Oikos.

[B6121052] Warwick R. M, Platt H. M., Somerfield P. J. (1998). Free-living marine nematodes. Part III. Monhysterids.

[B6121060] Wieser W. (1953). Die Bezichung swischen Mundhhlengestalt, Ernhrungsweise und Vorkommen bei freilebenden marinen Nematoden. Arkiv för Zoologi.

[B6121104] Wu X., Bezerra T. N.C., Gansbeke D., Moens T. (2019). Natural stable isotope ratios and fatty acid profiles of estuarine tidal flat nematodes reveal very limited niche overlap among co-occurring species. PeerJ.

[B6121113] Zhang Z. (2013). Animal biodiversity: An update of classification and diversity in 2013. Zootaxa.

