# Supplementary material for: Diversity patterns of free-living nematode assemblages in seagrass beds from the Cuban archipelago (Caribbean Sea)
Source: Biodivers Data J. 2020 Dec 23;8:e58848. doi: 10.3897/BDJ.8.e58848 (PMC7773714; doi:10.3897/BDJ.8.e58848)
Supplement: Supplementary material 2 — Counts of species of marine nematodes in 13 sites of seagrass beds from Cuban archipelago [file bdj-08-e58848-s002.docx]

**ESM1. Electronic Supplementary Material.** Counts of species of marine nematodes in 13 sites of seagrass bedss from Cuban archipelago.

| Species | GG | RG | SM | ON | OS1 | OS2 | AM1 | AM2 | AM3 | AM4 | GB1 | GB2 | GB3 |
| --- | --- | --- | --- | --- | --- | --- | --- | --- | --- | --- | --- | --- | --- |
| *Acanthonchus rostratus* Wieser, 1959 | 0 | 0 | 0 | 1 | 0 | 0 | 0 | 0 | 0 | 0 | 0 | 0 | 1 |
| *Acanthopharynx rigida* Schuurmans Stekhoven, 1950 | 0 | 0 | 18 | 1 | 3 | 0 | 0 | 0 | 0 | 0 | 0 | 0 | 0 |
| *Acanthopharynx sp.* | 0 | 0 | 1 | 0 | 0 | 0 | 0 | 0 | 0 | 0 | 0 | 0 | 0 |
| *Acanthopharynx viviparus* | 0 | 0 | 1 | 0 | 0 | 0 | 0 | 0 | 0 | 0 | 0 | 0 | 0 |
| *Actinonema pachydermatum* Cobb, 1920 | 0 | 0 | 0 | 0 | 0 | 0 | 0 | 0 | 0 | 1 | 0 | 0 | 0 |
| *Actinonema sp.* Cobb, 1920 | 0 | 0 | 4 | 0 | 0 | 0 | 0 | 0 | 0 | 0 | 0 | 0 | 0 |
| *Adoncholaimus sp.* Filipjev, 1918 | 0 | 0 | 0 | 0 | 0 | 0 | 1 | 0 | 0 | 0 | 0 | 0 | 0 |
| *Aegialoalaimus sp.* de Man, 1907 | 0 | 0 | 0 | 0 | 1 | 2 | 0 | 0 | 0 | 0 | 0 | 0 | 4 |
| *Anticoma sp.* Bastian, 1865 | 1 | 4 | 1 | 0 | 3 | 3 | 1 | 0 | 0 | 0 | 4 | 3 | 3 |
| *Anticoma trichura* Cobb, 1898 | 0 | 3 | 0 | 0 | 13 | 0 | 0 | 0 | 0 | 0 | 1 | 0 | 0 |
| *Bathyeurystomina sp.* Lambshead & Platt, 1979 | 0 | 1 | 0 | 0 | 0 | 0 | 0 | 1 | 0 | 0 | 0 | 0 | 0 |
| *Bolbonema brevicolle* Cobb, 1920 | 0 | 0 | 1 | 0 | 0 | 0 | 0 | 0 | 0 | 0 | 0 | 0 | 0 |
| *Catanema dambayensis* Tchesunov, 2013 | 0 | 0 | 2 | 0 | 0 | 0 | 0 | 0 | 0 | 0 | 0 | 0 | 0 |
| *Cephalanticoma chitwoodi* (Inglis, 1964) | 0 | 0 | 0 | 0 | 1 | 0 | 0 | 0 | 0 | 0 | 6 | 2 | 6 |
| *Ceramonema reticulatum* Chitwood, 1936 | 0 | 0 | 0 | 0 | 0 | 0 | 0 | 1 | 0 | 3 | 0 | 0 | 0 |
| *Ceramonema sp.* | 2 | 2 | 0 | 0 | 0 | 0 | 0 | 0 | 0 | 0 | 0 | 0 | 0 |
| *Chaetonema sp.* | 0 | 0 | 0 | 0 | 0 | 0 | 0 | 0 | 0 | 0 | 0 | 0 | 1 |
| *Cheironchus sp.* Cobb, 1917 | 0 | 3 | 0 | 0 | 3 | 0 | 2 | 2 | 0 | 0 | 0 | 0 | 2 |
| *Cheironchus vorax* Cobb, 1917 | 1 | 0 | 0 | 0 | 2 | 2 | 1 | 5 | 1 | 1 | 2 | 1 | 10 |
| *Chromadorella filiformis* (Bastian, 1865) | 0 | 0 | 1 | 2 | 0 | 0 | 0 | 0 | 0 | 0 | 0 | 0 | 0 |
| *Chromadorella sp.* Filipjev, 1918 | 0 | 0 | 0 | 2 | 2 | 0 | 0 | 0 | 0 | 0 | 2 | 0 | 0 |
| *Chromadorita sp.* Filipjev, 1922 | 0 | 0 | 0 | 0 | 0 | 0 | 0 | 0 | 0 | 0 | 0 | 0 | 1 |
| *Chromaspirina sp.* | 0 | 0 | 1 | 0 | 0 | 2 | 0 | 0 | 0 | 0 | 0 | 0 | 0 |
| *Cobbia sp.* | 0 | 4 | 0 | 0 | 0 | 0 | 0 | 0 | 0 | 1 | 0 | 0 | 2 |
| *Comesoma arenae* Gerlach, 1956 | 0 | 0 | 0 | 0 | 0 | 0 | 0 | 0 | 0 | 4 | 0 | 0 | 0 |
| *Comesoma sp.* | 0 | 0 | 2 | 0 | 0 | 0 | 0 | 2 | 0 | 0 | 0 | 1 | 0 |
| *Coninckia sp.* | 0 | 0 | 0 | 0 | 0 | 0 | 1 | 0 | 0 | 0 | 0 | 0 | 0 |
| *Crenopharynx marioni* (Southern, 1914) | 0 | 0 | 0 | 1 | 1 | 0 | 1 | 5 | 1 | 0 | 3 | 0 | 6 |
| *Crenopharynx paralepturus* (Schuurmans Stekhoven, 1950) | 0 | 0 | 0 | 0 | 0 | 0 | 0 | 0 | 0 | 0 | 2 | 0 | 4 |
| *Crenopharynx sp.* | 0 | 0 | 1 | 0 | 0 | 0 | 0 | 0 | 0 | 0 | 0 | 0 | 0 |
| *Croconema cinctum* Cobb, 1920 | 0 | 1 | 7 | 0 | 0 | 0 | 2 | 1 | 1 | 0 | 0 | 1 | 7 |
| *Cylicolaimus magnus* (Villot, 1875) | 0 | 2 | 0 | 13 | 1 | 0 | 2 | 1 | 0 | 1 | 0 | 0 | 0 |
| *Dagda bipapillata* Southern, 1914 | 0 | 0 | 1 | 0 | 0 | 0 | 0 | 1 | 0 | 0 | 0 | 0 | 1 |
| *Daptonema longicaudatum* (Filipjev, 1922) | 0 | 2 | 0 | 0 | 0 | 0 | 0 | 0 | 0 | 1 | 0 | 0 | 0 |
| *Daptonema oxycerca* (de Man, 1888) | 0 | 3 | 0 | 2 | 4 | 0 | 2 | 0 | 1 | 0 | 0 | 0 | 0 |
| *Daptonema sp.* | 0 | 4 | 1 | 0 | 4 | 38 | 6 | 1 | 6 | 3 | 0 | 5 | 11 |
| *Demonema rapax* Cobb, 1894 | 0 | 0 | 0 | 1 | 2 | 0 | 1 | 0 | 0 | 3 | 0 | 4 | 1 |
| *Demonema sp.* | 0 | 0 | 0 | 0 | 1 | 1 | 0 | 0 | 0 | 0 | 3 | 3 | 1 |
| *Desmodora pilosa* Ditlevsen, 1926 | 1 | 2 | 0 | 0 | 3 | 0 | 0 | 2 | 0 | 0 | 0 | 0 | 0 |
| *Desmodora pontica* (Filipjev, 1922) | 1 | 18 | 0 | 5 | 30 | 1 | 5 | 16 | 2 | 0 | 11 | 7 | 1 |
| *Desmodora scaldensis* de Man, 1889 | 1 | 2 | 1 | 0 | 2 | 0 | 1 | 8 | 1 | 0 | 0 | 0 | 1 |
| *Desmodora sp.* | 0 | 1 | 1 | 0 | 0 | 0 | 4 | 1 | 0 | 0 | 0 | 0 | 0 |
| *Desmoscolex spp.* | 1 | 1 | 0 | 0 | 13 | 0 | 12 | 7 | 9 | 4 | 1 | 0 | 2 |
| *Dichromadora apapillata* Timm, 1961 | 0 | 0 | 0 | 0 | 0 | 0 | 0 | 1 | 0 | 1 | 0 | 0 | 0 |
| *Dichromadora sp.* | 0 | 0 | 0 | 1 | 1 | 0 | 2 | 0 | 2 | 2 | 0 | 0 | 2 |
| *Didelta maculatum* Cobb, 1920 | 0 | 0 | 0 | 2 | 0 | 0 | 0 | 0 | 0 | 0 | 5 | 0 | 0 |
| *Didelta scutatum* Wieser, 1956 | 0 | 1 | 0 | 0 | 1 | 0 | 0 | 0 | 0 | 0 | 0 | 0 | 0 |
| *Didelta scutellatum* Vitiello, 1969 | 2 | 0 | 0 | 0 | 1 | 0 | 0 | 1 | 7 | 6 | 12 | 0 | 12 |
| *Didelta sp.* | 0 | 6 | 0 | 0 | 2 | 0 | 0 | 0 | 0 | 0 | 9 | 5 | 3 |
| *Disconema longicaudatum* Vitiello, 1969 | 0 | 1 | 0 | 1 | 0 | 0 | 2 | 1 | 2 | 1 | 1 | 0 | 3 |
| *Dolicholaimus sp.* | 1 | 0 | 0 | 0 | 0 | 0 | 0 | 0 | 0 | 0 | 0 | 0 | 0 |
| *Dorylaimopsis punctata* Ditlevsen, 1918 | 0 | 0 | 0 | 0 | 9 | 0 | 0 | 7 | 6 | 3 | 24 | 14 | 25 |
| *Dorylaimopsis sp.* | 0 | 0 | 0 | 0 | 4 | 0 | 0 | 12 | 10 | 0 | 3 | 0 | 0 |
| *Draconema sp.* | 0 | 0 | 4 | 0 | 0 | 0 | 0 | 0 | 0 | 0 | 0 | 0 | 0 |
| *Elzalia floresi* Gerlach, 1957 | 0 | 0 | 0 | 0 | 0 | 0 | 0 | 1 | 0 | 0 | 0 | 2 | 0 |
| *Elzalia sp.* | 1 | 1 | 0 | 0 | 0 | 0 | 0 | 0 | 0 | 0 | 6 | 2 | 0 |
| *Endeolophos minutus* (Gerlach, 1967) | 0 | 1 | 0 | 0 | 0 | 0 | 0 | 1 | 0 | 0 | 1 | 0 | 0 |
| *Enoploides sp.* | 0 | 17 | 0 | 3 | 2 | 2 | 2 | 0 | 0 | 0 | 1 | 0 | 0 |
| *Enoplolaimus mus* Inglis, 1964 | 0 | 0 | 0 | 4 | 0 | 0 | 0 | 0 | 0 | 0 | 0 | 0 | 0 |
| *Enoplolaimus sp.* de Man, 1893 | 0 | 2 | 0 | 1 | 0 | 1 | 1 | 1 | 1 | 2 | 0 | 0 | 1 |
| *Enoplus sp.* | 0 | 1 | 0 | 0 | 0 | 0 | 0 | 0 | 0 | 1 | 2 | 0 | 0 |
| *Epacanthion georgei* Inglis, 1971 | 0 | 1 | 0 | 0 | 0 | 0 | 0 | 0 | 0 | 0 | 0 | 0 | 0 |
| *Epsilonema spp.* | 0 | 0 | 7 | 0 | 0 | 0 | 0 | 0 | 0 | 0 | 0 | 0 | 0 |
| *Eubostrichus hopperi* Muthumbi, Verschelde & Vincx, 1995 | 2 | 0 | 7 | 0 | 1 | 0 | 0 | 0 | 0 | 0 | 0 | 1 | 0 |
| *Eubostrichus sp.* | 0 | 0 | 2 | 0 | 0 | 0 | 0 | 1 | 1 | 0 | 0 | 0 | 0 |
| *Euchromadora gaulica* Inglis, 1962 | 1 | 0 | 0 | 0 | 3 | 0 | 8 | 2 | 9 | 1 | 0 | 0 | 6 |
| *Euchromadora sp.* | 0 | 0 | 0 | 0 | 2 | 0 | 2 | 0 | 1 | 0 | 0 | 1 | 0 |
| *Euchromadora vulgaris* (Bastian, 1865) de Man, 1886 | 2 | 0 | 2 | 0 | 3 | 0 | 8 | 7 | 11 | 8 | 1 | 3 | 26 |
| *Eurystomina sp.* | 0 | 0 | 0 | 0 | 0 | 0 | 0 | 1 | 0 | 0 | 1 | 0 | 1 |
| *Filoncholaimus prolatus* Hopper, 1967 | 1 | 0 | 1 | 0 | 0 | 0 | 0 | 0 | 0 | 0 | 1 | 0 | 9 |
| *Gomphionema typicum* Wieser & Hopper, 1966 | 1 | 0 | 18 | 0 | 0 | 0 | 0 | 0 | 0 | 0 | 0 | 0 | 0 |
| *Graphonema sp.* | 0 | 0 | 0 | 0 | 0 | 0 | 0 | 0 | 1 | 0 | 0 | 0 | 0 |
| *Halalaimus bulbocaudatus* Keppner, 1992 | 0 | 0 | 0 | 0 | 0 | 0 | 0 | 0 | 0 | 0 | 0 | 0 | 1 |
| *Halalaimus filicollis* Timm, 1961 | 0 | 0 | 0 | 0 | 0 | 0 | 0 | 0 | 0 | 0 | 0 | 0 | 1 |
| *Halichoanolaimus chordiurus* Gerlach, 1955 | 0 | 3 | 2 | 3 | 3 | 0 | 0 | 14 | 15 | 3 | 5 | 15 | 5 |
| *Halichoanolaimus dolichurus* Ssaweljev, 1912 | 0 | 0 | 0 | 0 | 0 | 0 | 0 | 0 | 0 | 0 | 0 | 0 | 4 |
| *Halichoanolaimus duodecimpapillatus* Timm, 1954 | 0 | 0 | 0 | 1 | 1 | 0 | 0 | 0 | 1 | 0 | 5 | 1 | 1 |
| *Halichoanolaimus quattuordecimpapillatus* Chitwood, 1951 | 0 | 0 | 0 | 0 | 0 | 0 | 0 | 0 | 0 | 6 | 0 | 0 | 5 |
| *Halichoanolaimus sp.* | 0 | 4 | 1 | 1 | 15 | 1 | 0 | 0 | 1 | 2 | 15 | 4 | 22 |
| *Haliplectus sp.* | 0 | 1 | 0 | 0 | 1 | 0 | 0 | 0 | 0 | 0 | 0 | 0 | 0 |
| *Hopperia sp.* | 4 | 0 | 0 | 0 | 0 | 0 | 0 | 0 | 0 | 0 | 0 | 0 | 0 |
| *Hypodontolaimus sp.* | 0 | 0 | 4 | 0 | 0 | 0 | 0 | 0 | 0 | 1 | 0 | 0 | 0 |
| *Intasia nojii* (Jensen, 1991) | 0 | 0 | 1 | 1 | 0 | 0 | 0 | 0 | 0 | 0 | 0 | 0 | 0 |
| *Laimella longicauda* Cobb, 1920 | 1 | 2 | 0 | 0 | 0 | 4 | 0 | 0 | 0 | 0 | 0 | 1 | 0 |
| *Latronema sp.* | 2 | 11 | 0 | 1 | 0 | 0 | 0 | 15 | 4 | 4 | 0 | 0 | 2 |
| *Laxus cosmopolitus* Ott, 1995 | 0 | 0 | 0 | 0 | 0 | 0 | 1 | 0 | 1 | 0 | 0 | 0 | 0 |
| *Laxus parvum* Armenteros, Ruiz-Abierno & Decraemer, 2014 | 7 | 1 | 0 | 0 | 0 | 0 | 0 | 0 | 0 | 1 | 0 | 1 | 0 |
| *Laxus sp.* | 1 | 0 | 0 | 0 | 0 | 0 | 1 | 0 | 0 | 0 | 0 | 0 | 1 |
| *Leptolaimus vinnulus* Vitiello, 1974 | 0 | 0 | 4 | 0 | 0 | 0 | 0 | 0 | 0 | 0 | 0 | 0 | 0 |
| *Leptonemella brevipharynx* Armenteros, Ruiz-Abierno & Decraemer, 2014 | 6 | 2 | 0 | 0 | 1 | 0 | 1 | 2 | 1 | 0 | 0 | 0 | 0 |
| *Leptosomatum sp.* | 0 | 0 | 0 | 0 | 0 | 0 | 1 | 0 | 0 | 1 | 0 | 0 | 2 |
| *Linhomoeus elongatus* Bastian, 1865 | 0 | 0 | 6 | 0 | 9 | 19 | 0 | 0 | 2 | 1 | 1 | 0 | 17 |
| *Linhomoeus sp.* | 1 | 1 | 5 | 0 | 0 | 0 | 2 | 4 | 3 | 1 | 2 | 0 | 3 |
| *Linhystera problematica* Juario, 1974 | 0 | 0 | 0 | 0 | 0 | 0 | 0 | 0 | 2 | 0 | 0 | 0 | 0 |
| *Longicyatholaimus egregius* Hopper, 1972 | 5 | 7 | 1 | 4 | 0 | 0 | 0 | 0 | 0 | 0 | 0 | 1 | 2 |
| *Longicyatholaimus sp.* | 1 | 0 | 0 | 0 | 0 | 0 | 1 | 0 | 0 | 0 | 1 | 1 | 6 |
| *Lyranema sp.* | 0 | 1 | 0 | 4 | 12 | 0 | 0 | 0 | 0 | 0 | 0 | 2 | 0 |
| *Marylynnia annae* (Wieser & Hopper, 1967) | 0 | 2 | 0 | 0 | 0 | 0 | 0 | 0 | 0 | 0 | 3 | 3 | 0 |
| *Marylynnia eratos* (Hopper, 1972) | 2 | 1 | 0 | 1 | 0 | 0 | 1 | 0 | 0 | 0 | 0 | 0 | 0 |
| *Marylynnia sp.* | 7 | 21 | 1 | 7 | 3 | 0 | 4 | 2 | 7 | 3 | 5 | 3 | 16 |
| *Mesacanthion sp.* | 0 | 6 | 0 | 3 | 3 | 1 | 9 | 6 | 6 | 2 | 7 | 0 | 5 |
| *Metachromadora pulvinata* Wieser & Hopper, 1967 | 2 | 0 | 0 | 0 | 0 | 0 | 0 | 0 | 0 | 0 | 1 | 0 | 0 |
| *Metachromadora sp.* | 0 | 0 | 1 | 0 | 0 | 0 | 0 | 0 | 0 | 0 | 0 | 0 | 0 |
| *Metacomesoma sp.* | 0 | 0 | 0 | 0 | 0 | 0 | 0 | 0 | 0 | 0 | 0 | 0 | 1 |
| *Metacyatholaimus chabaudi* Gourbault, 1980 | 0 | 0 | 0 | 0 | 0 | 0 | 1 | 0 | 0 | 0 | 0 | 0 | 0 |
| *Metacyatholaimus effilatus* de Bovee, 1974 | 0 | 1 | 0 | 0 | 0 | 0 | 0 | 0 | 0 | 3 | 0 | 0 | 5 |
| *Metadasynemella sp.* | 0 | 0 | 0 | 0 | 0 | 0 | 0 | 2 | 0 | 0 | 0 | 0 | 0 |
| *Metadesmolaimus sp.* | 0 | 0 | 0 | 0 | 1 | 0 | 0 | 0 | 0 | 0 | 0 | 1 | 0 |
| *Metalinhomoeus biformis* Juario, 1974 | 0 | 0 | 0 | 0 | 0 | 0 | 0 | 0 | 1 | 0 | 1 | 0 | 0 |
| *Metalinhomoeus effilatus* Schuurmans Stekhoven, 1942 | 0 | 0 | 0 | 0 | 0 | 0 | 0 | 0 | 1 | 0 | 0 | 0 | 1 |
| *Metalinhomoeus sp.* | 0 | 0 | 0 | 1 | 7 | 4 | 1 | 1 | 0 | 1 | 0 | 0 | 0 |
| *Metalinhomoeus variabilis* Murphy, 1965 | 2 | 0 | 0 | 0 | 0 | 0 | 0 | 0 | 0 | 0 | 0 | 0 | 0 |
| *Micoletzkyia magna* Vitiello, 1970 | 0 | 0 | 0 | 0 | 1 | 0 | 0 | 0 | 0 | 0 | 0 | 0 | 0 |
| *Microlaimus sp.* | 2 | 9 | 0 | 0 | 1 | 0 | 0 | 0 | 0 | 0 | 0 | 0 | 0 |
| *Monoposthia mirabilis* Schulz, 1932 | 0 | 2 | 2 | 0 | 0 | 0 | 0 | 0 | 0 | 0 | 0 | 0 | 0 |
| *Neochromadora nicolae* Vincx, 1986 | 0 | 0 | 0 | 0 | 0 | 0 | 6 | 22 | 0 | 0 | 0 | 0 | 2 |
| *Odontophora bermudensis* Jensen & Gerlach, 1976 | 0 | 0 | 0 | 0 | 0 | 0 | 0 | 2 | 1 | 0 | 0 | 0 | 0 |
| *Odontophora sp.* | 0 | 3 | 0 | 0 | 0 | 0 | 0 | 3 | 1 | 0 | 0 | 0 | 0 |
| *Oxyonchus sp.* | 0 | 0 | 0 | 6 | 0 | 1 | 0 | 1 | 0 | 0 | 0 | 0 | 0 |
| *Oxystomina sp.* | 0 | 0 | 0 | 0 | 0 | 0 | 0 | 1 | 0 | 0 | 0 | 1 | 1 |
| *Paracanthonchus longicaudatus* Warwick, 1971 | 0 | 0 | 0 | 0 | 0 | 0 | 1 | 0 | 0 | 0 | 0 | 0 | 0 |
| *Paracomesoma dubium* (Filipjev, 1918) | 2 | 0 | 0 | 0 | 0 | 0 | 10 | 0 | 0 | 0 | 0 | 0 | 0 |
| *Paracomesoma sp.* | 1 | 0 | 0 | 0 | 0 | 0 | 1 | 1 | 0 | 0 | 0 | 0 | 0 |
| *Paradesmodora immersa* Wieser, 1954 | 4 | 36 | 7 | 2 | 18 | 1 | 10 | 13 | 7 | 0 | 2 | 1 | 7 |
| *Paradesmodora sinuosa* Ott, 1972 | 0 | 0 | 4 | 0 | 0 | 0 | 0 | 0 | 0 | 0 | 0 | 0 | 0 |
| *Paradesmodora sp.* | 0 | 1 | 0 | 0 | 0 | 0 | 3 | 0 | 0 | 0 | 0 | 0 | 0 |
| *Paralongicyatholaimus sp.* | 1 | 3 | 0 | 0 | 0 | 0 | 1 | 0 | 0 | 1 | 0 | 0 | 1 |
| *Paramesacanthion sp.* | 0 | 0 | 0 | 0 | 1 | 2 | 0 | 0 | 0 | 0 | 0 | 0 | 0 |
| *Paramesonchium sp.* | 0 | 0 | 0 | 0 | 0 | 0 | 0 | 0 | 0 | 1 | 0 | 0 | 0 |
| *Paramonohystera proteus* Wieser, 1956 | 0 | 0 | 0 | 0 | 0 | 0 | 2 | 0 | 0 | 0 | 0 | 1 | 0 |
| *Paramonohystera sp.* | 0 | 1 | 0 | 0 | 0 | 0 | 0 | 0 | 2 | 3 | 0 | 0 | 1 |
| *Paranticoma sp.* | 0 | 0 | 0 | 0 | 1 | 1 | 0 | 0 | 0 | 0 | 0 | 0 | 0 |
| *Pareurystomina sp.* | 0 | 0 | 0 | 0 | 0 | 0 | 0 | 0 | 0 | 0 | 0 | 0 | 1 |
| *Parodontophora cobbi* (Timm, 1952) | 0 | 0 | 0 | 0 | 0 | 0 | 0 | 2 | 1 | 1 | 0 | 0 | 0 |
| *Parodontophora sp.* | 0 | 0 | 1 | 0 | 0 | 0 | 0 | 1 | 1 | 0 | 0 | 0 | 2 |
| *Paroxystomina micoletzkyi* Wieser, 1953 | 0 | 0 | 0 | 2 | 0 | 0 | 0 | 0 | 0 | 0 | 0 | 0 | 0 |
| *Paroxystomina sp.* | 2 | 0 | 0 | 0 | 0 | 0 | 0 | 0 | 0 | 0 | 0 | 0 | 0 |
| *Perspiria hamata* Wieser & Hopper, 1967 | 0 | 0 | 0 | 0 | 0 | 0 | 0 | 0 | 0 | 0 | 0 | 0 | 3 |
| *Perspiria sp.* | 0 | 0 | 0 | 0 | 0 | 0 | 0 | 0 | 0 | 0 | 0 | 0 | 2 |
| *Phanoderma laticolle* (Marion, 1870) | 0 | 0 | 0 | 0 | 0 | 0 | 0 | 2 | 1 | 0 | 1 | 0 | 0 |
| *Phanoderma sp.* | 1 | 0 | 2 | 0 | 1 | 0 | 0 | 0 | 0 | 6 | 6 | 2 | 5 |
| *Phanodermella flagellicaudata* Vitiello, 1970 | 0 | 1 | 0 | 0 | 0 | 0 | 0 | 0 | 0 | 5 | 0 | 0 | 4 |
| *Phanodermopsis longisetae* Chitwood, 1936 | 0 | 0 | 0 | 0 | 0 | 0 | 0 | 2 | 0 | 1 | 0 | 0 | 0 |
| *Phanodermopsis sp.* | 0 | 0 | 0 | 0 | 0 | 0 | 1 | 0 | 2 | 0 | 0 | 0 | 1 |
| *Pierrickia sp.* | 0 | 0 | 0 | 0 | 0 | 0 | 0 | 1 | 0 | 1 | 0 | 0 | 0 |
| *Platycoma sp.* | 0 | 0 | 0 | 0 | 0 | 0 | 0 | 0 | 0 | 0 | 0 | 1 | 0 |
| *Platycomopsis sp.* | 0 | 0 | 0 | 0 | 0 | 0 | 1 | 0 | 0 | 0 | 0 | 0 | 0 |
| *Polygastrophora maior* Schulz, 1932 | 0 | 0 | 0 | 0 | 1 | 0 | 8 | 3 | 1 | 13 | 5 | 0 | 7 |
| *Polygastrophora sp.* | 0 | 0 | 0 | 0 | 0 | 0 | 0 | 1 | 0 | 1 | 0 | 0 | 2 |
| *Pomponema clavicaudatum* (Schuurmans Stekhoven, 1935) | 0 | 0 | 0 | 0 | 0 | 0 | 1 | 0 | 4 | 3 | 0 | 0 | 1 |
| *Pomponema concinnum* (Wieser, 1954) | 0 | 2 | 0 | 0 | 0 | 0 | 0 | 0 | 0 | 0 | 0 | 0 | 0 |
| *Pomponema sp.* | 3 | 0 | 0 | 0 | 0 | 1 | 2 | 1 | 1 | 1 | 1 | 0 | 3 |
| *Pontonema problematicum* Chitwood, 1960 | 0 | 0 | 2 | 0 | 0 | 0 | 0 | 0 | 0 | 0 | 0 | 0 | 0 |
| *Prochromadorella ditlevseni* (de Man, 1922) | 0 | 0 | 0 | 0 | 0 | 0 | 1 | 0 | 0 | 0 | 0 | 0 | 0 |
| *Promonhystera faber* Wieser, 1956 | 0 | 0 | 0 | 0 | 0 | 1 | 0 | 0 | 0 | 0 | 0 | 0 | 1 |
| *Promonhystera sp.* | 0 | 0 | 0 | 0 | 1 | 0 | 0 | 0 | 0 | 0 | 0 | 0 | 1 |
| *Pselionema simile* De Coninck, 1942 | 1 | 2 | 0 | 0 | 0 | 0 | 0 | 0 | 0 | 4 | 2 | 2 | 1 |
| *Pselionema sp.* | 4 | 8 | 0 | 0 | 0 | 2 | 0 | 0 | 0 | 0 | 3 | 8 | 1 |
| *Pseudosteineria sp.* | 0 | 2 | 1 | 0 | 0 | 0 | 0 | 0 | 0 | 2 | 0 | 0 | 0 |
| *Ptycholaimellus sp.* | 0 | 0 | 0 | 0 | 0 | 0 | 0 | 0 | 0 | 2 | 0 | 0 | 0 |
| *Rhinema retrorsum* Cobb, 1920 | 0 | 0 | 0 | 0 | 1 | 1 | 0 | 0 | 0 | 0 | 0 | 0 | 0 |
| *Rhinema sp.* | 0 | 2 | 0 | 0 | 0 | 1 | 0 | 0 | 0 | 0 | 0 | 0 | 0 |
| *Rhips anoxybiotica* Jensen, 1985 | 0 | 13 | 0 | 0 | 0 | 0 | 0 | 0 | 0 | 0 | 0 | 0 | 0 |
| *Rhips paraornata* Platt & Zhang, 1982 | 0 | 26 | 0 | 0 | 0 | 0 | 0 | 4 | 0 | 0 | 0 | 0 | 2 |
| *Rhips sp.* | 0 | 24 | 0 | 1 | 0 | 0 | 0 | 1 | 0 | 2 | 0 | 0 | 2 |
| *Richtersia coomansi* Soetaert & Vincx, 1987 | 0 | 0 | 0 | 0 | 0 | 0 | 0 | 1 | 0 | 0 | 0 | 0 | 5 |
| *Robbea porosum* (Hopper & Cefalu, 1973) | 2 | 0 | 1 | 0 | 0 | 0 | 1 | 0 | 0 | 0 | 0 | 0 | 0 |
| *Robbea sp.* | 1 | 0 | 0 | 0 | 0 | 0 | 0 | 4 | 0 | 0 | 0 | 0 | 0 |
| *Sabatieria armata* Gerlach, 1952 | 0 | 0 | 0 | 0 | 0 | 0 | 0 | 2 | 0 | 0 | 0 | 0 | 0 |
| *Sabatieria sp.* | 1 | 0 | 0 | 0 | 1 | 0 | 0 | 7 | 9 | 5 | 2 | 9 | 6 |
| *Scaptrella cincta* Cobb, 1917 | 1 | 0 | 0 | 0 | 0 | 0 | 0 | 1 | 0 | 0 | 1 | 0 | 0 |
| *Setoplectus sp.* | 0 | 0 | 0 | 2 | 0 | 0 | 0 | 0 | 0 | 0 | 0 | 0 | 0 |
| *Setosabatieria hilarula* (de Man, 1922) | 0 | 0 | 0 | 2 | 0 | 0 | 1 | 5 | 3 | 2 | 4 | 7 | 3 |
| *Setosabatieria sp.* | 0 | 0 | 0 | 1 | 1 | 0 | 0 | 0 | 0 | 0 | 1 | 0 | 0 |
| *Siphonolaimus sp.* | 0 | 0 | 0 | 0 | 0 | 0 | 1 | 0 | 0 | 1 | 0 | 0 | 0 |
| *Sphaerolaimus maeoticus* Filipjev, 1922 | 0 | 0 | 0 | 0 | 0 | 0 | 0 | 0 | 1 | 0 | 0 | 0 | 0 |
| *Sphaerolaimus sp.* | 0 | 1 | 0 | 0 | 0 | 11 | 0 | 0 | 0 | 0 | 0 | 0 | 0 |
| *Spiliphera sp.* | 0 | 2 | 0 | 0 | 0 | 0 | 0 | 0 | 0 | 0 | 0 | 0 | 0 |
| *Spilophorella candida* Gerlach, 1951 | 0 | 0 | 0 | 0 | 0 | 0 | 2 | 0 | 0 | 0 | 0 | 0 | 0 |
| *Spilophorella paradoxa* (de Man, 1888) | 0 | 1 | 0 | 0 | 0 | 0 | 1 | 1 | 2 | 5 | 0 | 1 | 1 |
| *Spilophorella sp.* | 1 | 1 | 1 | 0 | 0 | 0 | 0 | 0 | 0 | 2 | 0 | 1 | 0 |
| *Spirinia parasitifera* (Bastian, 1865) | 0 | 0 | 53 | 2 | 0 | 0 | 0 | 3 | 0 | 0 | 0 | 0 | 8 |
| *Spirinia sp.* | 0 | 1 | 5 | 0 | 1 | 0 | 1 | 0 | 4 | 0 | 0 | 0 | 3 |
| *Spirobolbolaimus bathyalis* Soetaert & Vincx, 1988 | 0 | 1 | 0 | 0 | 0 | 0 | 0 | 0 | 0 | 0 | 0 | 0 | 0 |
| *Steineria sterreri* Ott, 1977 | 0 | 0 | 0 | 1 | 0 | 0 | 0 | 0 | 0 | 0 | 0 | 0 | 0 |
| *Stilbonema brevicolle* Cobb, 1920 | 2 | 1 | 0 | 0 | 3 | 0 | 11 | 5 | 1 | 0 | 0 | 0 | 1 |
| *Stilbonema sp.* | 0 | 0 | 0 | 0 | 1 | 0 | 0 | 0 | 0 | 0 | 0 | 0 | 0 |
| *Symplocostoma tenuicolle* (Eberth, 1863) | 0 | 2 | 0 | 0 | 0 | 1 | 5 | 1 | 1 | 1 | 3 | 6 | 0 |
| *Synodontium sp.* | 0 | 0 | 0 | 0 | 0 | 0 | 0 | 1 | 0 | 0 | 0 | 0 | 0 |
| *Synonchiella micramphis* (Schuurmans Stekhoven, 1950) | 0 | 0 | 0 | 0 | 2 | 0 | 2 | 1 | 0 | 0 | 0 | 0 | 1 |
| *Synonchiella riemanni* Warwick, 1970 | 1 | 0 | 0 | 0 | 1 | 1 | 0 | 0 | 0 | 0 | 1 | 0 | 3 |
| *Synonchiella sp.* | 0 | 0 | 0 | 0 | 8 | 1 | 0 | 0 | 0 | 0 | 1 | 0 | 6 |
| *Synonchium sp.* | 1 | 0 | 0 | 0 | 1 | 0 | 0 | 0 | 1 | 0 | 1 | 0 | 0 |
| *Synonchus sp.* | 0 | 0 | 0 | 1 | 0 | 0 | 0 | 1 | 0 | 0 | 0 | 0 | 0 |
| *Syringolaimus filicaudatus* Vitiello, 1970 | 0 | 0 | 0 | 0 | 0 | 0 | 0 | 0 | 0 | 0 | 0 | 2 | 0 |
| *Syringolaimus striatocaudatus* de Man, 1888 | 0 | 0 | 3 | 0 | 0 | 0 | 0 | 0 | 0 | 0 | 0 | 0 | 0 |
| *Tarvaia sp.* | 0 | 0 | 0 | 0 | 0 | 0 | 1 | 0 | 0 | 0 | 0 | 0 | 1 |
| *Terschellingia gourbaultae* Austen, 1989 | 0 | 0 | 0 | 0 | 1 | 0 | 0 | 0 | 1 | 0 | 0 | 0 | 0 |
| *Terschellingia longicaudata* de Man, 1907 | 1 | 0 | 0 | 0 | 6 | 19 | 0 | 3 | 9 | 0 | 10 | 4 | 7 |
| *Terschellingia sp.* | 0 | 0 | 0 | 0 | 0 | 0 | 0 | 1 | 2 | 0 | 0 | 0 | 0 |
| *Thalassironus britannicus* De Man, 1889 | 0 | 0 | 0 | 5 | 1 | 0 | 0 | 0 | 0 | 0 | 0 | 2 | 0 |
| *Thalassironus sp.* | 3 | 2 | 0 | 1 | 0 | 0 | 0 | 1 | 0 | 2 | 0 | 0 | 4 |
| *Theristus otoplanobius* Gerlach, 1951 | 0 | 0 | 0 | 10 | 0 | 0 | 0 | 0 | 0 | 0 | 0 | 0 | 0 |
| *Theristus sp.* | 0 | 0 | 0 | 2 | 0 | 0 | 0 | 1 | 0 | 0 | 0 | 0 | 0 |
| *Tricoma spp.* | 0 | 0 | 0 | 1 | 7 | 0 | 0 | 1 | 0 | 0 | 4 | 4 | 0 |
| *Trileptium otti* Jensen & Gerlach, 1976 | 0 | 0 | 0 | 4 | 0 | 0 | 0 | 0 | 0 | 0 | 0 | 0 | 0 |
| *Trissonchulus janetae* Inglis, 1961 | 0 | 0 | 0 | 0 | 0 | 1 | 0 | 0 | 0 | 0 | 0 | 0 | 0 |
| *Trochamus carinatus* Boucher & de Bovée, 1971 | 0 | 0 | 0 | 2 | 0 | 0 | 0 | 0 | 0 | 0 | 0 | 0 | 0 |
| *Viscosia abyssorum* (Allgén, 1933) | 5 | 0 | 2 | 2 | 3 | 3 | 41 | 11 | 1 | 13 | 6 | 2 | 3 |
| *Viscosia glabra* (Bastian, 1865) | 5 | 0 | 4 | 0 | 1 | 0 | 8 | 1 | 4 | 3 | 2 | 1 | 4 |
| *Viscosia sp.* | 2 | 2 | 0 | 1 | 2 | 0 | 0 | 0 | 0 | 0 | 1 | 4 | 1 |
| *Xennella sp.* | 0 | 0 | 0 | 0 | 0 | 0 | 0 | 0 | 0 | 0 | 0 | 0 | 1 |
| *Xyala sp.* | 0 | 7 | 0 | 0 | 0 | 0 | 0 | 4 | 0 | 0 | 0 | 0 | 0 |
| *Zalonema ditlevseni* (Micoletzky, 1922) | 0 | 3 | 19 | 3 | 9 | 2 | 1 | 1 | 1 | 1 | 11 | 10 | 6 |
| *Zalonema sp.* | 0 | 0 | 1 | 0 | 0 | 0 | 0 | 0 | 0 | 0 | 0 | 0 | 0 |
